# Salt-Tolerant Plant Growth Promoting Rhizobacteria for Enhancing Crop Productivity of Saline Soils

**DOI:** 10.3389/fmicb.2019.02791

**Published:** 2019-12-18

**Authors:** Dilfuza Egamberdieva, Stephan Wirth, Sonoko Dorothea Bellingrath-Kimura, Jitendra Mishra, Naveen K. Arora

**Affiliations:** ^1^CAS Key Laboratory of Biogeography and Bioresource in Arid Land, Xinjiang Institute of Ecology and Geography, Ürümqi, China; ^2^Leibniz Centre for Agricultural Landscape Research (ZALF), Müncheberg, Germany; ^3^Faculty of Biology, National University of Uzbekistan, Tashkent, Uzbekistan; ^4^DST-CPR, Babasaheb Bhimrao Ambedkar University, Lucknow, India; ^5^Department of Environmental Science, Babasaheb Bhimrao Ambedkar University, Lucknow, India

**Keywords:** salinity, climate change, PGPR, crop productivity, agro-ecosystem

## Abstract

Soil salinity has emerged as a serious issue for global food security. It is estimated that currently about 62 million hectares or 20 percent of the world’s irrigated land is affected by salinity. The deposition of an excess amount of soluble salt in cultivable land directly affects crop yields. The uptake of high amount of salt inhibits diverse physiological and metabolic processes of plants even impacting their survival. The conventional methods of reclamation of saline soil which involve scraping, flushing, leaching or adding an amendment (e.g., gypsum, CaCl_2_, etc.) are of limited success and also adversely affect the agro-ecosystems. In this context, developing sustainable methods which increase the productivity of saline soil without harming the environment are necessary. Since long, breeding of salt-tolerant plants and development of salt-resistant crop varieties have also been tried, but these and aforesaid conventional approaches are not able to solve the problem. Salt tolerance and dependence are the characteristics of some microbes. Salt-tolerant microbes can survive in osmotic and ionic stress. Various genera of salt-tolerant plant growth promoting rhizobacteria (ST-PGPR) have been isolated from extreme alkaline, saline, and sodic soils. Many of them are also known to mitigate various biotic and abiotic stresses in plants. In the last few years, potential PGPR enhancing the productivity of plants facing salt-stress have been researched upon suggesting that ST-PGPR can be exploited for the reclamation of saline agro-ecosystems. In this review, ST-PGPR and their potential in enhancing the productivity of saline agro-ecosystems will be discussed. Apart from this, PGPR mediated mechanisms of salt tolerance in different crop plants and future research trends of using ST-PGPR for reclamation of saline soils will also be highlighted.

## Introduction

Since the inception of agricultural practices, the question that how to enhance crop productivity to feed the growing population has been challenging. As highlighted in 2018 Global Agricultural Productivity (GAP) Index the current growth rate of agricultural production is not enough to meet the projected food demand of 10 billion people in 2050 (GAP Report, 2018). The report also stated that under such circumstances GAP must be increased by 1.75% annually. Enhancing crop productivity in agro-ecosystems is intricate and greatly influenced by pedo-climatic conditions, farming systems and management techniques (Nemecek and Gaillard, 2010). A number of abiotic factors including temperature, salinity, drought, pesticides and fertilizer application, soil pH, and heavy metal contamination also hamper crop productivity (Ahmad, 2014). Amongst all these, salinization of arable land is being considered as a real menace for agricultural production. Recently, the United Nations’ Food and Agriculture Organization (FAO and ITPS, 2015) report “The status of the world’s soil resources” has identified nine major threats to soil functions and soil salinization is one of them. Soil salinization is globally expanding and in the past few years, the accelerated rate of salinization has also created food insecurity in several countries. The delta regions of India, Myanmar and Bangladesh which majorly contribute in world rice production are facing serious threats to food security due to salinization of coastal soil (Abedin et al., 2014; Szabo et al., 2016). According to Ghassemi et al. (1995) salt-affected, irrigated areas caused annual income losses in terms of $12 billion globally. In the United States, the central California region is losing approximately $3.7 billion in agricultural crop yield every year due to salinity (Dove, 2017). In Alberta, western province of Canada, on an average, 25 percent of crop yields are reduced annually due to salinity. Sindh region of Pakistan is suffering 31 percent crop loss annually due to waterlogging and salinity (Ilyas, 2017). These are just a few examples of this global menace.

In excess, any form of salt is deleterious for plant health. Soils with high salinity levels interfere with plants’ physiological process. The high salt concentration adversely affects important soil processes such as respiration, residue decomposition, nitrification, denitrification, soil biodiversity and microbial activity (Schirawski and Perlin, 2018). The loss of crop productivity and high salinity is also noticed where fertilizer input is too high in soil (Rütting et al., 2018). Application of high salt index fertilizers impose an osmotic effect which causes difficulty in extraction of water required for plant growth (Herger et al., 2015). It has also been observed that farming practices also affect crop production in saline soils. In such cases plowing down or tilling deeper increases the evaporation of water from the soil surface and deposit more salts. Apart from this, salts present in irrigation water may also increase soil salinity that results in productivity loss (Rengasamy, 2010; Arora et al., 2018). Earlier it was estimated that around 20 to 50 percent of irrigated lands worldwide were salt-affected (Szabolcs, 1992). It has also been projected that a huge proportion of the globe i.e., 24% or 60 million ha is being affected due to inappropriate irrigation practices (WWAP, 2012). Removal of salt from saline soil is an intensive process and requires too much time and money (Qadir et al., 2014). However, since long, reclamation of saline soils is mainly performed by physical and chemical processes. In a physical process, soluble salts in the root zone are removed by scraping, flushing and leaching methods (Ayyam et al., 2019). However, in chemical methods use of gypsum and lime as neutralizing agents is commonly done (Keren, 2005). But these methods are not sustainable and also considered inefficient when the salt concentration is too high.

Planting salt-tolerant crop varieties such as barley and canola on saline soils is common practice (Fita et al., 2015). However, due to normal salt tolerance profile, these crops have limited global reach and can’t be used in the soil with moderate or high electrical conductivity (EC) levels. Morton et al. (2019) also highlighted that despite vigorous efforts from the research community, only few salt tolerance genes have been identified having real applications in improving productivity of saline soils.

Hence, securing attainable crop yield in saline soils is the need of the hour and aside from using salt-tolerant varieties or amelioration methods involving chemical neutralizers, sustainable approaches should also be employed. In the last few years, research showed that the use of salt-tolerant plant growth promoting rhizobacteria (ST-PGPR) in saline agriculture can be harnessed for enhancing productivity and improving soil fertility as well (Grover et al., 2011). Their adaptive responses toward salt stress are related to the ability to produce osmoprotectants, compatible solutes, and specialized transporters. ST-PGPR are now being used as bioinoculants for enhancing crop yields, protection from phytopathogens and improving soil health. The present review targets the use of ST-PGPR for improving the productivity of crops grown in salt-affected soil. Their application in the form of bioinoculants for enhancing crop yield is also targeted. Apart from this, ST-PGPR mediated mechanisms, including the new insights and perspectives on productivity enhancement of crops facing the salinity stress are also discussed.

## Saline Soils: Global Distribution

**FIGURE 1:**
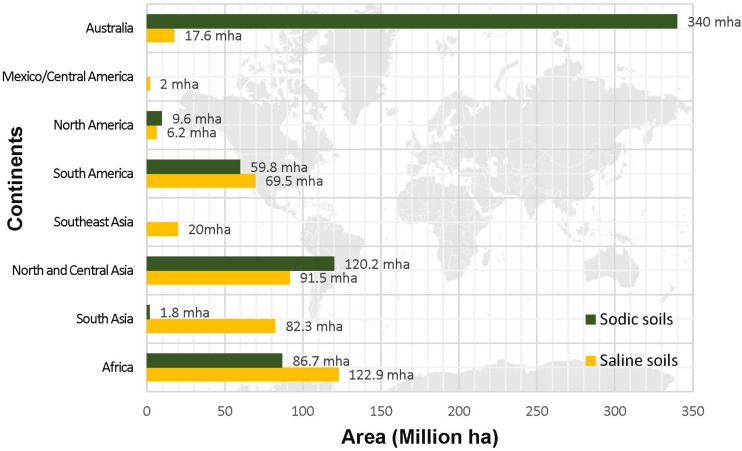
Global distribution of saline and sodic soil (Source: FAO and ITPS, 2015).

Although, it has been realized that all continents on the globe are facing the problem of soil salinity (Figure 1) yet accurate estimation of the locations and distribution of saline soils is missing. The Food and Agriculture Organization (FAO), United Nations Educational, Scientific and Cultural Organization –United Nations Environment Programme (UNESCO-UNEP), and International Society of Soil Science (ISSS) are the leading world agencies that paid attention in gathering data on quality of soil across the globe. The Soil Map of the World (FAO, 1971–1981) documented that a total area of 953 Million hectares (Mha) is salt-affected. According to FAO report on “Status of the World’s Soil Resources” soil of more than 100 countries with an estimated area of approximately one billion hectares is afflicted with the problem of salinity (FAO and ITPS, 2015). Due to very high amount of soluble salts (NaCl and Na_2_SO_4_) the EC of these soils exceeds 4 dSm^–1^. Currently, the soil classification system is governed by the World Reference Base for Soil Resources (WRB) which is endorsed by the International Union of Soil Sciences (IUSS) and it replaced the FAO/UNESCO Legend for the Soil Map of the World. The legend of the soil map described salt-affected soil as solonchak and solonetz. Solonchaks are characterized by the accumulation of high soluble salts. The salic horizon starts within ≤50 cm from the soil surface and are largely distributed to the arid and semi-arid coastal regions in all climatic zones in the world. The estimated global area of solonchaks is around 260 million hectares (IUSS Working Group WRB, 2015). According to national soil classification systems, solonchaks belong to Halomorphic Soils (Russia), Halosols (China) and Salids (United States of America) and EC may range from 8 to 15 dSm^–1^. On the basis of salt precipitation, there may be external solonchaks (deposition at the surface) or internal solonchaks (deposition at depth). Whereas solonetz which are commonly known as alkaline soils and sodic soils contain a large proportion of adsorbed Na and in some cases Mg ions also. However, solonetz with free Na_2_O_3_ are considered highly alkaline and their pH is recorded to be higher than 8.5. Globally, they are distributed in the semi-arid temperate continental climate. Area-wide, a total of 135 Mha solonetz are found in Ukraine, the Russian Federation, Kazakhstan, Hungary, Bulgaria, Romania, China, the United States of America, Canada, South Africa, Argentina, and Australia (IUSS Working Group WRB, 2015).

## The Diversity of Salt-Tolerant Plant Growth Promoting Rhizobacteria (St-Pgpr)

Soil has a huge versatility of microorganisms, belonging to different groups of bacteria, fungi, and archaea. Amongst microbes, some are now well known for their inherent capability to tolerate different concentrations of salt and to promote plant growth as well. These salt-tolerant plant beneficial microbes have great importance in agriculture. They have shown their potential in improving crop productivity in arid and semiarid regions (Niu et al., 2018). The genera *Pseudomonas*, *Bacillus*, *Enterobacter*, *Agrobacterium*, *Streptomyces*, *Klebsiella*, and *Ochromobacter* are best reported for improving the productivity of diverse crops under saline conditions (Sharma et al., 2016; Singh and Jha, 2016; Sarkar et al., 2018). The diazotrophic salt-tolerant bacterial strains of *Klebsiella*, *Agrobacterium*, *Pseudomonas*, and *Ochrobactrum* isolated from the roots of a halophytic plant, *Arthrocnemum indicum* showed salinity tolerance ranging from 4 to 8% NaCl, and improved the productivity of peanut in saline as well as in control conditions (Sharma et al., 2016). *Planococcus rifietoensis*, an alkaliphilic bacterium is reported to enhance growth and yield of wheat crop under salinity stress (Rajput et al., 2013). Upadhyay et al. (2009) explored the genetic diversity of ST-PGPR isolated from the wheat rhizosphere. They found that most of the isolates were able to tolerate up to 8% NaCl and belong to the genus *Bacillus*. The diversity of salt-tolerant bacteria isolated from paddy rhizosphere in Taoyuan, China was reported by Zhang et al. (2018). They isolated 305 bacterial strains, and amongst them, 162 were tested for salt tolerance up to 150 g/l NaCl concentration. Phylogenic analysis of 74 of these salt-tolerant strains showed that they belong to orders Bacillales (72%), Actinomycetales (22%), Rhizobiales (1%), and Oceanospirillales (4%). Most of the isolates also showed their potential in improving salt tolerance, growth, and yield of rice under salt-stress conditions. ST-PGPR strain *Bacillus licheniformis* SA03 isolated from *Chrysanthemum* plants grown in saline-alkaline soil of China conferred increased salt tolerance in *Chrysanthemum* (Zhou et al., 2017).

The diversity of salt-tolerant bacilli was also deciphered in the soil of eastern Indo Gangetic plains of India by Sharma et al. (2015). They isolated 95 bacterial strains and amongst them, 55 showed plant growth promoting characteristics and salt-tolerance to more than 4% NaCl. Several researchers also report the diversity of ST-PGPR in the coastal areas. For example, in Tsunami affected regions in Andaman and Nicobar Islands of India, 121 bacterial strains were isolated, and amongst them 23 showed salt tolerance up to 10% NaCl with PGP characteristics including production of indole acetic acid (IAA), siderophore, extracellular enzymes and phosphate solubilization (Amaresan et al., 2016). The study revealed that the majority of isolates were *Bacillus* spp. and rest were *Alcaligenes faecalis*, *Microbacterium resistance*, *Enterobacter* sp., *Lysinibacillus* sp. A recent study claimed the presence of a novel salt-tolerant bacterial strain *Pseudomonas* sp. M30-35 from the rhizosphere of *Haloxylon ammodendron*, a C_4_ perennial succulent xerohalophyte with outstanding drought and salt tolerance capabilities. *Pseudomonas* sp. M30-35 was found to contain 34 genes possessing homology with certain genes associated with PGP traits and abiotic stress tolerance (He et al., 2018). Recently, the genome of many ST-PGPR have also been sequenced which envisaged information about their salt tolerance and plant growth promoting attributes. Kothari et al. (2013) reported that *Bacillus safensis* VK from the desert of Gujrat, India, showed salt tolerance up to 14% NaCl and pH ranging from 4 to 8. Further, study of this *B. safensis* strain deciphered that its genome harbors several genes associated with PGP traits functioning in conditions of high salt concentrations, drought, heavy metals, and polyaromatic hydrocarbons (PAHs) contamination. ST-PGPR *Klebsiella* sp. IG 3 isolated from the rhizosphere of wheat showed salt tolerance up to 20%. This strain positively modulated the expression profile of *rbcL* (codes for the ribulose-1,5-bisphosphate carboxylase/oxygenase RuBisCo) and *WRKY1* (transcription factor dealing with plants reaction to biotic stress) genes under salt-stress conditions (Sapre et al., 2018). In another study, whole genome analysis of a halotolerant PGPR *Klebsiella* sp. D5A revealed the presence of salt tolerance genes with a wide range of pH adaptability and PGP traits including phosphate solubilization, IAA biosynthesis, acetoin, and 2,3-butanediol synthesis, siderophore production, and N_2_ fixation (Liu et al., 2016). *Pseudomonas putida* and *Novosphingobium* sp. are reported to reduce salt-stress induced damage in citrus plants by lowering the level of abscisic acid (ABA) and salicylic acid (SA), reducing the efficiency of photosystem II (Fv/Fm), increasing accumulation of IAA in the leaf and inhibiting accumulation of root chloride and proline during salt stress (Vives-Peris et al., 2018). A *Pseudomonas* strain isolated from halophilic grass *Distichlis spicata* also improved the growth of different crops under salt stress (Palacio-Rodríguez et al., 2017). A ST-PGPR strain *Enterobacter* sp. UPMR18 with ability to produce 1-aminocyclopropane-1-carboxylic acid (ACC) deaminase showed improvement in crop productivity through induction of reactive oxygen species (ROS) scavenging enzymes including superoxide dismutase (SOD), ascorbate peroxidase (APX) and catalase (CAT) and upregulating to ROS pathway genes (Habib et al., 2016a). Recently, functional metagenomics provided a magnificent way of identification of various genes responsible for salt resistance in microorganisms.

## Effect of Soil Salinization on Crops

**FIGURE 2:**
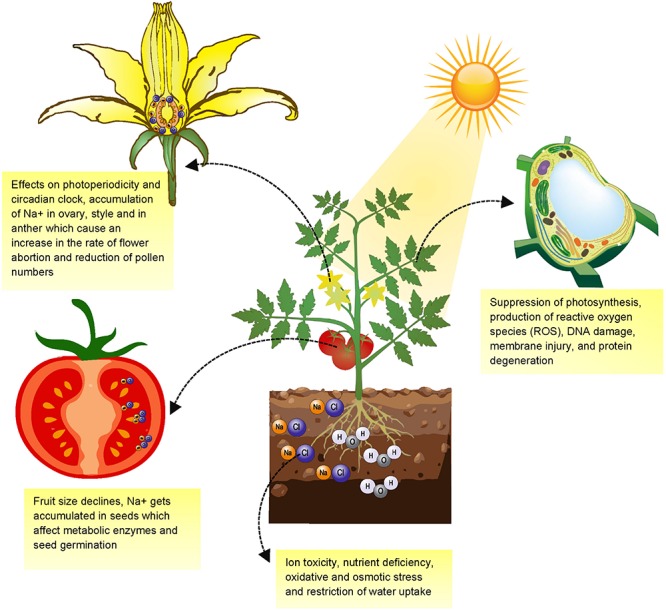
Effect of salinity stress on plant development.

Soil salinity has an overall detrimental effect on plants’ health (Figure 2). Salinity affects flowering and fruiting pattern, aberration in reproductive physiology, which ultimately influences crop yields and biomass. Salinity may cause up to 50% reduction in flowering of pigeon pea (*Cajanus cajan* L. Mill) (Promila and Kumar, 1982). In tomato, high salt stress (150 mm NaCl) is reported to affect flowering transition time and causes delay in the first inflorescence along with reduction in the growth of shoot and root (Ghanem et al., 2009). In chickpea (*Cicer arietinum* L.), delayed flowering was directly linked with the higher concentrations of Na^+^ in the laminae of completely expanded leaves (Pushpavalli et al., 2016). Salt Overly Sensitive (SOS) pathway is a major defense pathway involved in Na^+^ extrusion and maintaining ion homeostasis at the cellular level (Zhu et al., 1998; Zhu, 2003; Ji et al., 2013). There are several reports where both SOS and photoperiodical and circadian clock switch proteins related with flowering are deactivated by salt stress (Kim et al., 2013; Park et al., 2013, 2016; Ryu et al., 2014).

Salt stress remarkably affects plant reproductive physiology. Ghanem et al. (2009) reported that in tomato the exposure of salinity stress results in Na^+^ accumulation in style, ovaries, and anther intermediate layers which caused an increase in the rate of flower abortion, reduction of pollen number and viability of the plant. Läuchli and Grattan (2007) reported that salt stress decelerates reproductive growth of wheat by inhibiting spike development and decreasing the yield potential, whereas in salt-sensitive rice, lowering of yield by the reduction in tillers, and formation of sterile spikelet is also experienced. The effect of salinity on *Arabidopsis* was explored in hydroponic solution which induced many symptoms including reduced fertility, decline in fruit length, transient wilting and fruits with predominantly aborted ovules and embryos which were narrower and reduced in size (Sun et al., 2004). Similarly, the effect of salt on early flowering and male gametophyte of canola (*Brassica napus*) plant showed a reduction in pollen grain numbers and abnormal growth of anthers. All these symptoms indirectly lead to reduced crop yield (Mahmoodzadeh and Bemani, 2008).

The drastic effect of salt stress can be seen in terms of yield loss. The primary effects related to crop yield can be in terms of germination which either decreases or sometimes ceases under extreme saline conditions. A study by Ali Khan et al. (2012) showed that under saline conditions growth, yield, and biomass of pearl millet is adversely affected in terms of germination percentage, plant height, leaf area, total biomass and grain yield plant^–1^. Impact of salinity on pea was also found to adversely affect growth, yield and biomass (Wolde and Adamu, 2018). Farooq et al. (2017) also reviewed the effects of salt stress on grain legumes, and they described that in different legumes salinity may reduce crop yield by 12–100%. Salt tolerance of black cumin (*Nigella sativa* L.) and its effect on seed emergence and germination, and yield were studied by Faravani et al. (2013). They showed that an increase in salinity level from 0.3 to 9 dS m^–1^ reduced the average seed and biological yield. Similarly, the effect of different levels of salinity on a weed plant *Portulaca oleracea* L. which is of nutritional importance and being utilized in same ways as spinach and lettuce in many countries, showed a reduction in biomass and yield, changes in physiological attributes, and alteration in stem and root structure (Amirul Alam et al., 2015). Salinity has thus a wide level of impacts on different crops, including even complete loss of yields. This also has impact on soil quality, greenhouse gas (GHG) emissions and food security.

## Mechanisms of Pgpr Mediated Salt Stress Tolerance

**FIGURE 3:**
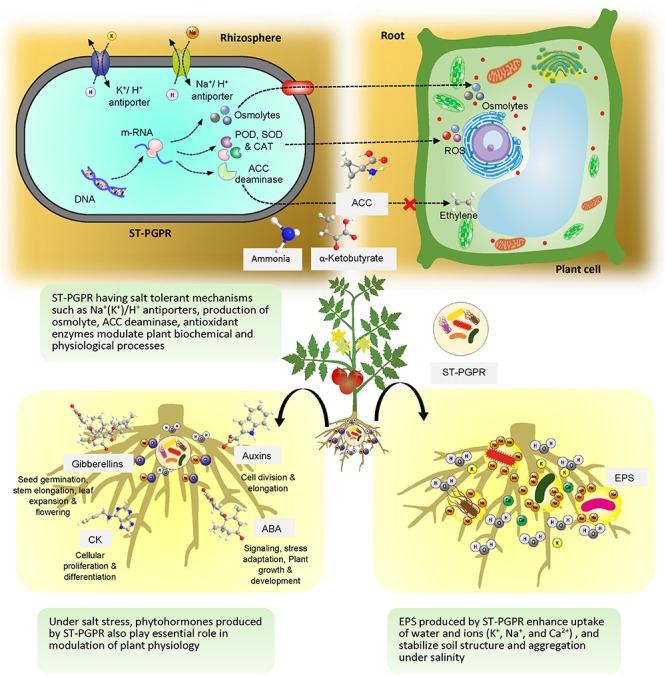
Salt-tolerant plant growth promoting rhizobacteria mediated mitigation of salt stress in plants.

The ST-PGPR utilize an array of mechanisms (Figure 3) which directly or indirectly take part in amelioration of salt stress in crop plants (Egamberdieva et al., 2016; Hashem et al., 2016). Studies confirmed that ST-PGPR produce various types of phytohormones, such as auxins, gibberellins, cytokinins (Dodd et al., 2010), synthesize ACC deaminase (Glick et al., 2007), produce secondary compounds such as exopolysaccharides (Upadhyay et al., 2012; Timmusk et al., 2014) and osmolytes (proline, trehalose, and glycine betaines) (Bano and Fatima, 2009; Upadhyay and Singh, 2015), regulate plant defense systems and activate plant’s antioxidative enzymes under salt stress (Hashem et al., 2016).

### Plant Growth Regulators

Phytohormones produced by ST-PGPR play an essential role in modulation of plant physiology under salt-stress (Egamberdieva and Kucharova, 2009). The ST-PGPR produce IAA which is required for cell division and elongation in plants coping with salt stress. Some of the best-known ST-PGPR producing IAA under salt stress are *Azotobacter, Arthrobacter*, *Azospirillum*, *Pseudomonas*, *Stenotrophomonas*, and *Rahnella* (Egamberdieva et al., 2008, 2018; Piccoli et al., 2011; Abd_Allah et al., 2017). Studies have confirmed that under salt stress yield loss in crops can be minimized by the application of phytohormone producing ST-PGPR. Under saline conditions, *P. putida* modulated IAA synthesis in plant tissue and increased the growth parameters of cotton (Yao et al., 2010). It has also been observed that inoculation of ST-PGPR increased the uptake of minerals, protected plants from ion toxicity and enhanced root and shoot growth under saline conditions (Egamberdieva et al., 2017a). Sadeghi et al. (2012) reported that salt-tolerant *Streptomyces* isolates with IAA producing ability improved the root system of wheat under salt stress. Recently, Kang et al. (2019) reported that IAA produced by a ST-PGPR *Leclercia adecarboxylata* MO1 has linkages with sugar synthesis, organic acid production and chlorophyll fluorescence improvement (Fv/Fm) in tomato. Apart from auxins other phytohormones are also reported to alleviate the effect of salt stress in plants. For example, production of cytokinins (CK), which are important for cellular proliferation and differentiation have been reported in salt-tolerant *Arthrobacter*, *Bacillus*, *Halomonas*, *Azospirillum*, and *Pseudomonas* species (García de Salamone et al., 2001; Karadeniz et al., 2006; Naz et al., 2009; TrParray et al., 2016). ABA is also synthesized by strains of ST-PGPR including *Proteus mirabilis*, *Bacillus megaterium*, *B. licheniformis*, *Pseudomonas fluorescens*, and *Achromobacter xylosoxidans* (Karadeniz et al., 2006; Forchetti et al., 2007; Salomon et al., 2014). Gibberellin producing bacterial strains such as *Bacillus pumilus*, *B. licheniformis*, *Azospirillium* sp. were also reported by Bottini et al. (2004). There are reports where ST-PGPR are known to produce more than one type of phytohormones. Patel and Saraf (2017) reported that bacterial strains of *Pseuodomonas stutzeri*, *Stenotrophomonas maltophilia*, and *P. putida* isolated from the *Coleus* rhizosphere produced IAA, gibberellic acid, and CK under saline conditions. Recently, Tewari and Arora (2018) reported the role of SA in ameliorating salinity stress and enhancing the growth of sunflower in saline soils.

### ACC Deaminase Activity

The presence of ACC deaminase activity in ST-PGPR is a common phenomenon especially when exposed to high salt stress. The enzyme ACC deaminase cleaves ACC into ammonia and α-ketobutyrate which are consumed by the bacteria as nitrogen (N) and carbon sources (Glick, 2014). The presence of ST-PGPR dramatically lowers the level of stress ethylene and prevents inhibition of down-regulated genes involved in ethylene-induced plant stress and up-regulates genes involved in plant growth (Glick et al., 2007). There are many examples where ACC deaminase activity by ST-PGPR not only improved plant survival in saline soils but also enhanced productivity. *Stenotrophomonas rhizophila* synthesized ACC deaminase and stimulated growth of cucumber in saline soil (Egamberdieva et al., 2011). Ali et al. (2014) reported improvement in physiological properties of plants under salt stress by ACC deaminase producing *P. fluorescens* and *Pseudomonas migulae* strains. In another study, halotolerant bacterial strains, *Brachybacterium saurashtrense* (JG-06), *Brevibacterium casei* (JG-08), and *Haererohalobacter* (JG-11), producing ACC deaminase, improved salt tolerance in peanut grown in saline soils (Shukla et al., 2012). It has been found that ACC deaminase producing ST-PGPR have effects on other biochemical properties of plant cell, including membrane stability, biocompatible solutes formation and photosynthetic pigment production under drought and salt stress (Tiwari et al., 2018). Aslam and Ali (2018) also reported that ACC-deaminase activity in halolerant bacterial genera of *Arthrobacter*, *Bacillus*, *Brevibacterium*, *Gracilibacillus*, *Virgibacillus*, *Salinicoccus*, and *Pseudomonas*, *Exiguobacterium* isolated from the rhizosphere and phytoplane of *Suaeda fruticosa* (L.) Forssk stimulated growth of maize under saline conditions. The effect of ACC deaminase by ST-PGPR on nodule formation in legume crops is also well documented (Ahmad et al., 2011; Barnawal et al., 2014). In nodulation process, ACC deaminase is found to play an essential role to enhance persistence of infection threads which is negatively affected by ethylene level and thus help in nodule formation under saline conditions (Nascimento et al., 2016).

### Osmoprotectants

Plants accumulate organic osmolytes such as proline, glycine, betaine, polyamines, quaternary ammonium compounds, and other amino acids in response to various abiotic stresses (Sandhya et al., 2010). ST-PGPR also employ this mechanism for protection against osmotic stress which is more common in saline soils (Mishra et al., 2018). While exposed to salt stress, salt-tolerant bacteria may temporarily increase their cytoplasmic content of K^+^, but the accumulation of osmolytes is a more sustained stress response to prevent water loss (Bremer and Kramer, 2019). During salt stress, internal concentration of organic osmolytes may reach up to 1 M in certain halophilic bacteria and has a major role in destabilization of the double helix and lower the Tm of DNA (Rajendrakumar et al., 1997). In moderately halophilic bacteria salinity induced expression of proline biosynthesis genes *proH*, *proJ*, and *proA* was reported at 2.5 M NaCl which lead to the highest accumulation of proline (Saum and Müller, 2007). Recently, Kushwaha et al. (2019) explored osmoprotection in *Halomonas* sp. SBS 10 and found that at low NaCl, betaine accumulation suppresses the *de novo* synthesis of ectoine whereas at a high NaCl concentration, the ectoine concentration increases abruptly as compared to the betaine. Further, they concluded that ectoine accumulation is transcriptionally up-regulated by the salinity stress. In another study it was observed that *Azospirillum* spp. accumulate proline, glycine betaine, and trehalose, supporting the plant to withstand osmotic stress (Rodríguez-Salazar et al., 2009). A ST-PGPR strain of *Bacillus* sp. increased growth and development of maize under drought and salinity through accumulation of proline and soluble sugars (Vardharajula et al., 2011). Role of the trehalose as an osmoprotectant under salt-stress is also well documented and a large number of ST-PGPR have been discovered having genes for trehalose biosynthetic pathways (Qin et al., 2018; Orozco-Mosqueda et al., 2019; Shim et al., 2019).

### Exopolysaccharides (EPS)

Production of EPS or surface polysaccharides is a general characteristic of some rhizosphere bacteria. Although, the composition and amount of EPS may vary in different ST-PGPR strains, copious amount of EPS is formed in adverse conditions (Bomfeti et al., 2011; Tewari and Arora, 2014a; Khan and Bano, 2019). EPS work as physical barrier around roots and support plant growth in high salinity stress (Vaishnav et al., 2016). Inoculation of EPS producing ST-PGPR also showed ameliorative effects on the uptake of K^+^, Na^+^, and Ca^2+^ in plants (Ashraf et al., 2004; Kohler et al., 2006). Qurashi and Sabri (2012a) showed that EPS producing ST-PGPR *Halomonas variabilis* (HT1) and *P. rifietoensis* (RT4) improved chickpea growth and stabilization of soil structure and aggregation under salinity. The presence of EPS in the biofilm also has positive effects on root colonization by ST-PGPR (Qurashi and Sabri, 2012b). In the context of yield improvement the role of EPS producing ST-PGPR is very significant as they are being used as priming agents of seeds and help in enhancing germination (Tewari and Arora, 2014a). Atouei et al. (2019) found that salt-tolerant EPS producing *B. subtilis* subsp. *inaquosorum* and *Marinobacter lipolyticus* SM19 reduced adverse effects of salinity and drought stresses in wheat. Recently, Chu et al. (2019) showed possible role of EPS producing salt-tolerant *Pseudomonas* PS01 strain in regulation of genes related to stress tolerance in *Arabidopsis thaliana*. They found that a LOX2 gene which encodes a lipoxygenase that constitutes an essential component of the jasmonic acid (JA) synthesis pathway was up-regulated. As JA itself is a positive regulator and accumulates rapidly in plants (under salt stress), bacterial EPS provide additional benefits to survive under salt stress.

### Antioxidant Enzymes

Inoculation of plants with ST-PGPR decreases the negative effects of oxidative stress by producing antioxidative enzymes (Manaf and Zayed, 2015; Islam et al., 2016). There are evidences where ST-PGPR produced high concentration of antioxidant enzymes including POD, SOD, CAT, and nitrate reductase (NR), glutathione reductase (GR) under salinity stress (Jha and Subramanian, 2013; Sen and Chandrasekhar, 2015; Ansari et al., 2019). El-Esawi et al. (2019) observed that *Azospirillum lipoferum* FK1 inoculated plants demonstrated a higher expression level of the antioxidant genes and thus improved antioxidant enzymes and non-enzymatic metabolites, nutrient uptake, phenols and flavonoids content, growth, and development of chickpea. Similarly, Kohler et al. (2009) observed an increase in CAT activity in tissue of lettuce, reducing oxidative damage under saline conditions. Some ST-PGPR such as *Enterobacter clocae*, *Pseudomonas pseudoalcaligenes* and *Bacillus* sp., increased levels of APX and CAT in Jatropha leaves in response to salt stress and also stimulated the roots, increased biomass, N, phosphorus (P), potassium (K) uptake and chlorophyll content in the vegetative parts of the plant (Patel and Saraf, 2013). The salt-tolerant plant *Sulla carnosa* showed better growth and stress tolerance after inoculation with strains of *Pseudomonas* sp. and *Bacillus* sp. in saline Tunisian soils. The bacterial inoculants increased root and shoot biomass, and nutrient acquisition under salt stress by reducing stomatal conductance, and modulated antioxidant activities involved in plant stress responses (Hidri et al., 2016).

Taken together, these findings clearly suggest the important role of ST-PGPR in plant stress tolerance by modulating plant physiological and biochemical processes such as stress-related genes, osmolytes, and enzymatic and non-enzymatic antioxidants.

## St-Pgpr for Improving Crop Productivity

It is now widely accepted that ST-PGPR are endowed with the inherent capability to cope with high concentration of salts in the soil. Their presence in the soil provides a direct benefit to plants. Their application in the form of bioinoculants/bioformulations not only improves crop productivity but also makes the survival of plants easier under extreme saline conditions (Table 1). In this section, the role of ST-PGPR in enhancing productivity of various crops is discussed.

**TABLE 1:** Salt-tolerant plant growth promoting rhizobacteria and their possible roles in enhancing yield and growth of diverse plants/crops.

**Crops**	**ST-PGPR**	**Mechanism**	**References**
Sunflower	*P. fluorescens*	IAA production, siderophore production and K^+^/Na^+^ ratio	Shilev et al., 2012
	*Pseudomonas aeruginosa*	EPS production	Tewari and Arora, 2014b
	*Pseudomonas* sp.	IAA production, phosphate solubilization, siderophore, nitrogen fixation, HCN, chitinase and β-1-3 glucanase activity	Tewari and Arora, 2016

Soybean	*P. putida* H-2-3	ABA, salicylic acid and JA and gibberellins	Kang et al., 2014
	*Bradyrhizobium japonicum* USDA 110 and *P. putida* TSAU1	Root system physiology, nitrogen and phosphorus acquisition and nodule formation	Egamberdieva et al., 2017a
	*Bacillus firmus* SW5	Antioxidant enzyme and alternation in root system architecture	El-Esawi et al., 2018a
	*Bradyrhizobium japonicum, Bacillus subtilis* SU-12 and *Serratia proteamaculans*	Antioxidant enzyme and Proline content	Han and Lee, 2005
	*Pseudomonas simiae*	IAA production, phosphate solubilization and siderophore production	Vaishnav et al., 2016
	*B. japonicum* and *B. subtilis*	EPS production, antioxidant activity and concentration of proline	Han and Lee, 2005
	*P. fluorescens*	CK production	Bhattacharyya and Jha, 2012

Groundnut	*P. aeruginosa* AMAAS57 and *P. aeruginosa* BM6	IAA production, HCN, ammonia, phosphate solubilization, production of phenol and free amino acids	Ghorai et al., 2015
	*B. saurashtrense, B. casei, Haererohalobacter*	Osmotic stress and proline	Shukla et al., 2012
	*P. fluorescens*	ACC deaminase	Saravanakumar and Samiyappan, 2007
	*Klebsiella, Pseudomonas, Agrobacterium*, and *Ochrobactrum*	IAA production, phosphate solubilization, ACC deaminase	Sharma et al., 2016

Rape seed	*Rhizobium* sp.	ACC deaminase, IAA production and phosphate solubilization	Saghafi et al., 2018
	*P. fluorescens* and *P. putida*	ACC deaminase, IAA and hydrogen cyanide	Jalili et al., 2009
	*P. putida* UW4	Photosynthesis, antioxidant enzyme, membrane transportation and pathogenesis-related responses	Cheng et al., 2012

Cotton seed	*P. putida R4* and *Pseudomonas chlororaphis* R5	IA production	Egamberdieva et al., 2015
	*P. putida*	Germination rate and biomass	Yao et al., 2010
	*Bacillus amyloliquefaciens, Curtobacterium oceanosedimentum* and *Pseudomonas oryzihabitans*	Seed germination	Irizarry and White, 2017
	*Klebsiella oxytoca* Rs-5, *Bacillus* sp. SL-13, *Bacillus* sp. SL-14 and *Bacillus* sp. SL-44	Antioxidant enzymes and photosynthetic pigment content	Wu et al., 2012

Rice	*Alcaligens* sp., *Bacillus* sp. and *Ochrobactrum* sp.	ACC deaminase	Bal et al., 2013
	*Serratia* sp. and *Pseudomonas* sp.	IAA production, nitrogen fixation, and phosphate solubilization	Nakbanpote et al., 2014
	*P. pseudoalcaligenes* and *B. pumilus*	Reduced the toxicity of ROS by reducing plant cell membrane index, cell caspase-like protease activity, and programmed cell death	Jha and Subramanian, 2013
	*Bacillus aryabhattai* MS3	Nitrogen fixation, IAA production, phosphorus solubilization and siderophore production	Sultana et al., 2018
	*Enterobacter* sp. P23	Phosphate solubilization, IAA production, siderophore production, HCN production	Sarkar et al., 2018
	*Halobacillus dabanensis* strain SB-26, *Halobacillus* sp. GSP 34	Nitrogen fixation and IAA production	Rima et al., 2018
	*Pseudomonas* strains PF1 and TDK1	Antioxidant enzyme	Sen and Chandrasekhar, 2015
	*B. stratosphericus* (NBRI 5Q and NBRI 7A)	Phosphate solubilization, ACC deaminase activity IAA production	Misra et al., 2017
	B. amyloliquefaciens NBRISN13 (SN13)	Betaine, sucrose and trehalose	Nautiyal et al., 2013
	*Bacillus* and *Citrobacter*	Nitrogen fixation, phosphate solubilization and IAA production	Habib et al., 2016b
	*B. pumilus*	Antioxidative enzymes	Khan et al., 2016

Chick pea	*P. pseudoalcaligens*	Phosphate solubilization, siderophore and IAA production	Patel et al., 2012
	*Mezorhizobium ciceri*	Nodulation and Nitrogen fixation	Egamberdieva et al., 2014
	*H. variabilis (HT1)* and *P. rifietoensis (RT4)*	Biofilm formation and EPS	Qurashi and Sabri, 2012a
	*Mesorhizobium strains*	ACC deaminase activity improved nodulation and reduced level of ethylene	Chaudhary and Sindhu, 2015

Mung bean	*Rhizobium* and *pseudomonas*	Photosynthetic rate, chlorophyll content and water use efficiency	Ahmad et al., 2013
	*Rhizobium* sp.	ACC deaminase	Aamir et al., 2013
	*P. fluorescens* (Mk20) and *Rhizobium phaseoli*	ACC deaminase	Ahmad et al., 2011

Lentil	*Rhizobium leguminosarum* bv.viciae	IAA production and phosphate solubilization	Jida and Assefa, 2011
	*Pseudomonas jessenii* and *R. leguminosarum* (P10Z22)	ACC deaminase	Zahir et al., 2011
	*Rhizobium* sp. BCKV MU5 and BCKV MU2	Nodulation efficiency	Halder et al., 2016

Pea	*Arthrobacter protophormiae* with *R. leguminosarum* and *Glomus mosseae*	Reduction of ethylene stress through ACC deaminase	Barnawal et al., 2014

Pigeon pea	*Bradyrhizobium* (RA-5) and *Burkholderia cepacia* (RRE-5)	Enhancement of root nodulation and N_2_ fixation	Bano et al., 2015
	*P. putida, P. fluorescens, Bacillus cereus with Rhizobium strain*	Nodulation and N_2_ fixation	Tilak et al., 2006

Black gram	*P. fluorescens* SA8 with kinetin (10 μM)	Improvement in water relation, gas exchange, and photosynthetic content	Yasin et al., 2018

Faba bean	*P. putida*, *P. fluorescens* and *B. subtilis*	Increase in growth traits of plant	Metwali et al., 2015

	*Pseudomonas* anguilliseptica SAW 24	Biofilm production and EPS production	Mohammed, 2018
Maize	*Pseudomonas syringae*, *Enterobacter aerogenes* and *P. fluorescens*	ACC deaminase	Nadeem et al., 2007
	*Azospirillum brasilense*	Ion toxicity, NOR and nitrogenase activity	Hamdia et al., 2004
	*Pseudomonas* and *Enterobacter* spp.	ACC deaminase	Nadeem et al., 2009
	*Azotobacter chroococcum*	Improved K^+^/Na^+^ratio, polyphenol content and proline concentration	Rojas-Tapias et al., 2012
	*Proteus penneri*, *P. aeruginosa*, and *A. faecalis*	EPS	Naseem and Bano, 2014
	*B. amyloliquefaciens*	Soluble sugar content and antioxidant enzymes	Chen et al., 2016
	*Pantoea agglomerans*	Up-regulation of aquaporin genes	Gond et al., 2015
	*Rhizobium* and *Pseudomonas*	Osmotic regulation	Bano and Fatima, 2009
	*Staphylococcus sciuri*	Antioxidant enzymes	Akram et al., 2016
	*Bacillus* spp. and *Arthrobacter pascens*	phosphate solubilization, osmotic regulation and antioxidant enzymes	Ullah and Bano, 2015
	*Bacillus aquimaris* DY-3	Chlorophyll content, osmotic regulation and antioxidant enzymes	Li and Jiang, 2017
	*P. syringae* and *P. fluorescens*	ACC deaminase	Zafar-ul-Hye et al., 2014
	*A. brasilense strains Ab-V5 and Ab-V6 and Rhizobium tropici strain CIAT 899*	Antioxidant enzymes and proline contents	Fukami et al., 2018
	*Gracilibacillus*, *Staphylococcus*, *Virgibacillus*, *Salinicoccus*, *Bacillus*, *Zhihengliuella*, *Brevibacterium*, *Oceanobacillus*, *Exiguobacterium*, *Pseudomonas*, *Arthrobacter*, and *Halomonas* spp.	IAA production, ACC deaminase, phosphate solubilization and biofilm formation	Aslam and Ali, 2018
	*Serratia liquefaciens* KM4	Facilitated gas exchange, osmoregulation, antioxidant enzymes, nutrient uptake and downregulation of ABA biosynthesis	El-Esawi et al., 2018b

### Cereal Crops

Cereal crops are the main source of energy and protein in the human diet. Worldwide, in comparison to other crops, cereal crops are cultivated in much higher quantity. Wheat, maize, rice, barley, oats, sorghum, and millet are known as major cereal crops. However, very few of them are reported as salt-tolerant. Since decades, most common approaches of enhancing yield of saline soils, which include conventional breeding, marker-assisted selection, and genetic engineering have also proven to be successful, but only for wheat and rice (Shahbaz and Ashraf, 2013; Roy et al., 2014). It has been realized that application of ST-PGPR in salt-affected soil not only assisted in the survival of crop but also improved yield in a wide range of cereal crops (Singh and Jha, 2016). In a study, ST-PGPR *B. subtilis* enhanced wheat yield by around 18% in salt-affected soil (EC 5.2 dSm^–1^) (Upadhyay and Singh, 2015). Research shows that application of ST-PGPR in rice may increase germination, promote seedling growth, induce antioxidative enzymes against ROS, favor osmolyte accumulation and modulate expression of genes related to salt stress (Nautiyal et al., 2013; Paul and Lade, 2014; Rima et al., 2018; Sarkar et al., 2018). The effect of various ST-PGPR in enhancing the productivity of salt-tolerant rice and wheat cultivated on sodic soils was explored by Damodaran et al. (2019) and they found that *Lysinibacillus* sp. was most effective in ameliorating the adverse effect of salinity. Similarly, a comprehensive research on the diversity of ST-PGPR in different agro-climatic zones was conducted by Misra et al. (2017) and they revealed that amongst all, *Bacillus* sp. with ACC deaminase activity were most dominant in amelioration of salt stress and enhancing biomass of rice. A ST-PGPR, *Pseudomonas* strain 002 was found to improve root formation in maize under salinity (150 mM NaCl) stress (Zerrouk et al., 2016). The effect of ST-PGPR *S. sciuri* SAT-17 strain on anti-oxidative defense mechanisms and modulation of maize growth under salt stress was studied by Akram et al. (2016). They reported that inoculation of maize with SAT-17 improved plant growth and decreased the ROS levels by increasing the cellular antioxidant enzyme activities (CAT, POD, and proline) under salinity treatments (75 and 150 mM NaCl).

### Legumes and Oil Yielding Crops

Along with the cereals, legumes reserve their position as important source of protein in the human diet. Salinity hampers production of grain and food legumes in many regions around the globe (Manchanda and Garg, 2008). In legumes, salt-stress adversely effects root-nodule formation, symbiotic relation and finally the nitrogen fixation capacity (Manchanda and Garg, 2008). Symbiotic association of rhizobia with legumes under salinity stress is still a broad area of research (Zahran, 1991, 1999; Graham, 1992). Most of the findings reveal that application of salt-tolerant rhizobia is a sustainable solution for enhancing the productivity of legume crops grown under salinity stress (Abiala et al., 2018). Some workers have demonstrated that negative impacts of salinity on legumes including soybean, pigeon pea, common bean, mung bean, groundnut and even tree legumes can be minimized by the application of salt-tolerant rhizobial strains (Bashan and Holguin, 1997; Kumar et al., 1999; Dobbelaere et al., 2001; Bashan et al., 2004; Dardanelli et al., 2008; Meena et al., 2017; Yasin et al., 2018). Salt-tolerant species of *Pseudomonas* are reported to improve the health of groundnut (Saravanakumar and Samiyappan, 2007), common beans (Egamberdieva, 2011), faba beans (Metwali et al., 2015), and chickpea (Jatan et al., 2019). In a study, application of ST-PGPR *B. firmus* SW5 on soybean grown under salt stress showed its beneficial effect on nutrient uptake, chlorophyll synthesis, osmolyte levels, gas exchange parameters, total phenolics, flavonoid contents, and antioxidant enzyme activities, in comparison to control (El-Esawi et al., 2018a). Wang et al. (2016) found that ACC-deaminase containing rhizobacterium *Variovorax paradoxus* 5C-2 increased total biomass of pea by 25 and 54% under salinity levels of 70 and 130mM NaCl, respectively. In a study, Yanni et al. (2016) used four strains of *Rhizobium* as bioinoculants for common bean in saline and drought stressed fields and found that inoculation increased seed yield considerably. A combined effect of *R. phaseoli* with L-tryptophan on the mung bean grown under salinity stress was evaluated by Zahir et al. (2010) and it was found that the application induced more pronounced effects and increased the plant height, number of nodules per plant, plant biomass, grain yield, and grain N concentration as compared with the untreated control. In another study, Ahmad et al. (2011) evaluated the combined application of salt-tolerant *Rhizobium* and *Pseudomonas* under salt-stressed conditions for improving the productivity of mung bean. They found that co-inoculation increased all the growth parameters including seed yield by 150% as compared with un-inoculated control. *B. japonicum* USDA 110 and *P. putida* TSAU1 were reported with salt tolerance activity and abilities to work synergistically with each other to enhance growth and productivity of soybean under high salinity (Egamberdieva et al., 2017b).

Salinity halts the growth and affects the oil yield in several legumes. Field application of ST- PGPR in oil-yielding crops has proven beneficial in remediation of salt stress (Tewari and Arora, 2014a,b). In other studies fluorescent *Pseudomonas* improved root and shoot length of sunflower under saline conditions (Tewari and Arora, 2016) and ST-PGPR *Klebsiella*, *Agrobacterium*, *Pseudomonas*, and *Ochrobactrum* enhanced salt-tolerance in groundnut crop (Sharma et al., 2016). Similarly, salt-tolerant *P. fluorescens* strain TDK1 with ACC deaminase activity also enhanced the salt resistance in groundnut plants, which in turn enhanced yields (Saravanakumar and Samiyappan, 2007). Furthermore, co-inoculation of *B. japonicum* USDA 110 and salt-tolerant *P. putida* TSAU1 were found to improve growth parameters, protein content, N, and P uptake and root system architecture of soybean under salt stress (Egamberdieva et al., 2017a).

### Vegetable Crops

Globally, about 1.1 percent of the total agricultural area is covered with vegetables^1^ and China and India are the leading countries in this regard. The threshold of salinity for most vegetable crops is ≤2.5 dS m^–1^ hence compared with others, vegetable crops are more prone to damage caused due to salinity stress (Shannon and Grieve, 1998). Unfortunately, research on the role of ST-PGPR in alleviating salt stress of vegetable crops is very scarce. However, there are some reports about the role of ST-PGPR in enhancing the performance of vegetable crops under salinity stress. Mayak et al. (2004) isolated an *Achromobacter piechaudii* bacterium from Arava region of southern Israel which significantly increased the fresh and dry weights of tomato seedlings at high salt concentration. Similarly, Tank and Saraf (2010) also found that the ST-PGPR *P. stutzeri* improved salt tolerance in tomato plants. Recently, Van Oosten et al. (2018) reported that inoculation of tomato plants with *A. chroococcum 76A* under both moderate (50 mM NaCl) and severe (100 mM NaCl) salt stresses increased growth parameters i.e., shoot dry weight, fresh fruit weight and fruit number per plant as compared to the control. Abd El-Azeem et al. (2012) reported that ST-PGPR strains of *Xanthobacter autotrophicus* BM13, *E. aerogenes* BM10, and *Bacillus brevis* FK2 alleviated negative effects of salt and improved growth and yield in egg plant. Furthermore, Ge and Zhang (2019) showed that under salt stress (3% NaCl), *Rhodopseudomonas palustris* G5 increased shoot height, root length, fresh weight, dry weight, total chlorophyll content and soluble sugar content of cucumber seedlings. Recently, Tahir et al. (2019) conferred that consortium of salt-tolerant *Bacillus* strains enhanced potato tuber yield by the production of auxin, antioxidant enzymes and regulating uptake of Na^+^, K^+^, and Ca^+2^ in normal and salt affected soils. ST-PGPR have proven to be beneficial for cultivation of vegetables in saline soils, however, this needs to be explored and utilized further.

## Future Prospects

Although much progress has been made in understanding the effect of salinity on plants, little success is achieved in the sustainable management of productivity losses. The potential of ST-PGPR can be harnessed for the improvement of crop yield of saline soils. According to Pan et al. (2019) physiological roles played by ST-PGPR could improve plant performance under saline conditions. It has been realized that elucidation of mechanisms of osmo-adaptation in ST-PGPR may contribute to the long-term goal of improvement of productivity in crops grown in saline agro-ecosystems (Paul, 2013). Knowledge of salt tolerance mechanisms in ST-PGPR is still not up to mark, particularly about the involvement of bacterial genes in osmotic regulation and plant-microbe interactions (under saline conditions). Although, in the last few years, molecular mechanisms of salt tolerance have been studied in several halophilic bacteria. This revealed that amongst various mechanisms, bacterial salt-related antiporters such as Na^+^/H^+^ play specific roles in salinity tolerance in plants as well (Zhong et al., 2012). Ma et al. (2019) stated that the understanding of regulatory networks of ST-PGPR in inducing salt tolerance in plants, could serve as a promising measure to alleviate salt stress and improve global food production. However, a deeper investigation of microbial responses to soil salinity are required for their better utilization in the reclamation of saline soils. Kim et al. (2019) suggested that identification of the dominant indigenous microflora from the highly saline soil and their possible adaptation mechanisms may provide a better understanding for exploring ecological and evolutionary responses in ecosystems. The role of metagenomic and metabolomic approaches becomes very important in case of harnessing and identifying novel ST-PGPR, along with the key genes and metabolites involved in salt tolerance.

Crop specific field trials (Table 1) have already suggested strong ability of ST-PGPR in mitigation of salt stress (in diverse crops). However, replication of these research findings with same vigor in different geographical regions and on different plants has remained a challenge. This is why, in some of the instances, erratic results are obtained while using PGPR inoculants at field level (Souza et al., 2015; Ambrosini et al., 2016). Recently, Kumar et al. (2019) suggested that salinity related research on PGPR should follow certain protocols and screening is an important issue to maintain the quality and accuracy. They also addressed that use of indigenous ST-PGPR strains should be preferred in bioinoculant development as they can easily adapt to the local field conditions. Similarly, development of ST-PGPR based bioinoculant exclusively designed for saline soils could be more efficient approach in managing productivity loss in several crops (Mishra et al., 2018). Whereas prospects on future research of developing novel bioinoculants providing metabolites (osmolytes, biosurfactants, precursor of phytohormones and stress enzymes) along with efficient ST-PGPR strains (including consortia) should also be explored. Consortia based bioinoculants have recently gained importance because by involving diverse ST-PGPR not only salinity stress can be managed but phytopathogens can also be controlled and nutrients be assimilated simultaneously (Woo and Pepe, 2018). It has also been suggested that use of the diverse microbes such as PGPR, mycorrhiza and endosymbionts in consortial formulations can be a very promising strategy for combating stress in plants (Ilangumaran and Smith, 2017). At present there is lack of products of ST-PGPR for specific use in saline agro-ecosystems and available bioinoculants do not work in such conditions. This makes the end-user skeptical about the use of biological alternatives. The use of metabolites such as EPS has already been suggested and explored in some latest bio-products (Tewari and Arora, 2014b; Arora and Mishra, 2016). EPS can be cheaply produced at lab and industrial levels and can be added to bioinoculants prepared for saline soils. This metabolite can act as protectant for both the inoculated PGPR and the plant. Similarly other metabolites and carriers can also be explored for development of tailor-made bioformulations for saline soils.

Nowadays, several researchers are vigorously engaged to enhance the efficacy and applicability of ST-PGPR containing bioinoculants in field conditions (Ambrosini et al., 2016). Furthermore, a major part of research has to be devoted to improve the issues related with formulation development (Mishra et al., 2018). In recent past, development in the field of polymeric science devised amalgamated use of biopolymers and PGPR in agriculture (Raj et al., 2011; Sharif et al., 2018). For example, chitosan-immobilized aggregated *Methylobacterium oryzae* CBMB20 has been used as a bioinoculant to promote growth of tomato under salt stress conditions (Chanratana et al., 2019). Li et al. (2019) also showed synergistic use of a Super Absorbent Polymer (SAP) along with PGPR strains of *Paenibacillus beijingensis* BJ-18 and *Bacillus* sp. L-56 to overcome salinity stress in wheat and cucumber. Enhancing the productivity of saline soils will not only help in achieving food security but also result in enhanced content and quality of soil organic matter in these nutritionally poor agro-systems. This can help in reducing the carbon footprint and combating climate change as well (Arora, 2019; SDG, 2019).

## Conclusion

Salt-tolerant plant growth promoting rhizobacteria have evolved several mechanisms to cope with salinity stress. They can easily withstand high salinity stress through various mechanisms such as efflux systems, formation and accumulation of compatible solutes for balancing external osmotic pressure, formation of ROS, secondary metabolites and other means. Several genes and metabolites are involved in maintaining the cell integrity and plant-microbe interactions under salinity stress. Still a lot is yet to be explored at molecular and biochemical level on how the ST-PGPR support themselves and their symbiotic partner under salinity stress which has multi-dimensional impacts on the cell (of both bacteria and plant). Research on ST-PGPR also indicates their vast potential in remediation and productivity enhancement of agro-ecosystems suffering from problems of salinity. However, in-depth studies targeting gene level expression and functional characteristics of ST-PGPR involved in plant growth promotion under salinity stress have to be conducted in the near future to design tailor-made bioformulations for saline soil systems, which are increasing worldwide, day by day. Utilization of this green biotechnology will have multi-faceted positive impacts on agro-ecosystems and rural environment.

## Author Contributions

DE and NA conceptualized the idea. JM and NA prepared the illustrations. All authors equally contributed in writing of the manuscript.

## Conflict of Interest

The authors declare that the research was conducted in the absence of any commercial or financial relationships that could be construed as a potential conflict of interest.

## Footnotes

1 http://www.fao.org/3/i3138e/i3138e05.pdf
